# GC/MS-Based Urine Metabolomics Study on the Ameliorative Effect of *Xanthoceras sorbifolia* Extract on Alzheimer's Disease in Mice

**DOI:** 10.1155/2022/3390034

**Published:** 2022-09-17

**Authors:** Rui Han, Min Wang, Li Wang, Yichen Zhang, Xin Li, Yijun Hou, Jing Yan, Xiaojing Pan

**Affiliations:** ^1^School of Stomatology, Lanzhou University, Donggang Road No. 199, Lanzhou 730020, China; ^2^School of Basic Medical Sciences, Lanzhou University, Donggang Road No. 199, Lanzhou 730020, China

## Abstract

The cause of Alzheimer's disease, the most common type of dementia today, is still unclear, and in current research, there are no drugs that work relatively well. Therefore, the study for new drugs to treat Alzheimer's disease is an urgent research need. Research on the improvement of Alzheimer's disease with extracts of *Xanthoceras sorbifolia* has been increasing in recent years, but the mechanism is not fully understood. The experiments were conducted to validate the model and analyze the treatment effect through D-galactose and A*β*_25–35_ induced dementia model mice, using the Morris water maze, to detect the learning behavior and brain tissue section to observe the hippocampal tissue structure of mice. We performed a nontargeted metabolomic analysis of the urine obtained from different groups of mice using gas chromatography-mass spectrometry. Fourteen potential biomarkers were identified in the mice's urine, outlining five metabolic pathways of interest. It was shown that the extracts of *Xanthoceras sorbifolia* may exert protective effects on mice in dementia models through energy metabolism, neuroinflammation, and antioxidants. This study reveals the potential pathogenesis of Alzheimer's disease and the possible therapeutic mechanism of *Xanthoceras sorbifolia*, suggests relevant biomarkers, and provides an additional basis for the clinical application of *Xanthoceras sorbifolia*.

## 1. Introduction

Alzheimer's disease (AD) is considered a neurodegenerative disease characterized by progressive, permanent memory impairment, and cognitive dysfunction [1]. AD can lead to memory loss, personality changes, impairment of speech, visual and spatial abilities, abnormal behavior, and even loss of self-care ability [1–3]. AD accounts for about 50–75% of all dementia patients and is the most common type of dementia [4]. The prevalence of AD increases with age, and the number of people with AD is expected to reach 131.5 million worldwide in 2050 [5]. Because there is no permanent treatment for AD, the World Health Organization (WHO) has declared AD a “global public health priority” [2]. The pathogenesis of AD is still unclear, and the recognized causes include acetylcholine deficiency, free radicals, and inflammation of brain tissue; and there is no cure, but effective drug therapy can improve the patients' symptoms to improve the quality of life [6–8]. Many medicines in traditional Chinese medicine can tonify the kidneys, which has a better effect on the treatment of AD [9]. A study comparing AD animal models treated with Chinese herbal medicine to those treated with donepezil showed no statistically significant efficacy. In contrast, Chinese herbs such as the heart-regulating formula consisting of *Radix Codonopsis*, *Poria*, *Polygala tenuifolia Willd*, *Calamus*, and *Cassia twig*, and the kidney-tonifying formula consisting of *Radix Rehmanniae*, *Ophiopogon japonicus*, *Cornu Cervi Pantotrichum*, and *Fructus Psoraleae*, showed a lower incidence of abnormal liver function [10].

*Xanthoceras sorbifolia* is a plant of the genus *Xanthoceras* native to northern China and is used as an oilseed plant and a traditional Chinese medicinal plant [11, 12]. Recently, the potential pharmacological effects of *Xanthoceras sorbifolia* extract on improving learning and memory disorders and the treatment of AD have received increasing attention [12–14]. Xanthoceraside is a widely reported triterpene with anti-AD effects. Animal studies have shown that xanthoceraside can improve learning and memory impairment by reducing neuroinflammatory responses, protecting synaptic morphology and dendritic spines, altering gut microbial metabolism levels, and affecting insulin signaling pathways [15–18]. However, the current research on the extracts of *Xanthoceras sorbifolia* is insufficient, so more in-depth studies on its anti-AD mechanism are needed.

Metabolomics is a powerful tool to study the changes of low molecular weight endogenous metabolites (≤1500 Da), including sugars, amino acids, organic acids, and lipids [19]. The metabolome, as the result of gene expression, protein modification, and environmental exposure, is a refinement and complement to biological information [20]. The pathophysiological process of AD has not been fully elucidated, and the application of metabolomics in AD research may help to elucidate the etiology and pathogenesis of AD further and provide new ways for the diagnosis and treatment of AD [21, 22]. Most of the current studies on AD metabolomics are based on cerebrospinal fluid, blood, or postmortem brain tissue samples, while urine metabolite analysis has been less studied [21]. However, the in vivo accumulation process of urine allows for the accumulation of metabolites that contain a more decadent amount of information compared with a single point in time blood or cerebrospinal fluid [23]. Moreover, urine can be obtained noninvasively, which can reduce the subjects' pain and is more beneficial for clinical applications. In this study, we used the gas chromatography/mass spectrometry (GC/MS) technique to reveal the differences in critical metabolites in mice's urine. We explored the potential mechanism of the extract of *Xanthoceras sorbifolia* on improving cognitive impairment in AD model mice.

## 2. Materials and Methods

### 2.1. Preparation of *Xanthoceras sorbifolia* Extract

*Xanthoceras sorbifolia* was obtained from Northwest *Xanthoceras sorbifolia* base (Baiyin, China). Dried *Xanthoceras sorbifolia* husks and hulled seed kernels were taken separately, crushed, sieved, and placed in a round bottom flask. Referring to related studies [24, 25], for the fruit husks, 70% ethanol solution was added at a stock-to-liquid ratio of 1 : 7 g/L, and the extracts were collected twice at 70°C in a water bath with heat reflux for 5 h each. For the seeds, petroleum ether was added at a ratio of 1 : 4 g/L, and the extracts were collected by refluxing the petroleum ether extracts twice at 60°C for two hours each time under the heat of a water bath. After that, the filtered residue of the petroleum ether extract was collected and dried, and then, 70% ethanol solution was added at a ratio of 1 : 7 g/L, and the extract was extracted twice at 70°C under the heat of water bath for five hours each time, and the extract was collected. After vacuum filtration, the extracts were combined. The solvent was recovered by rotary evaporation and dried to a constant weight, and the extraction rate was calculated and stored at −4°C for backup. Ginsenoside Re was obtained from Shanghai Yuanye Bio-Technology Co., Ltd (Shanghai, China). The content of total saponins in the extract was determined spectrophotometrically using Ginsenoside Re as the standard. The total saponin content of the ethanolic extract of the *Xanthoceras sorbifolia* husks was 69.75%, and the total saponin content of the petroleum ether extract of the seed kernel was 46.07%, as determined by the regression equation.

### 2.2. Drugs and Reagents

Amyloid beta-peptide 25–35 (A*β*_25–35_) was purchased from Shanghai Yien Chemical Technology Co., Ltd (Shanghai, China). D-galactose (Gal) was purchased from Solarbio Science & Technology Co., Ltd (Beijing, China). Donepezil was provided by Eisai Co., Ltd (Tokyo, Japan). Urease, ribitol, pyridine, methoxyamine hydrochloride, and BSTFA-TMCS (99 : 1) were purchased from G-Clone Biotechnology Co., Ltd (Beijing, China). Avertin was purchased from Nanjing Aibei Biotechnology Co., Ltd (Shanghai, China). The other analytical grade reagents were obtained from Sinopharm Chemical Reagent Co., Ltd (Shanghai, China).

### 2.3. Animals and Modelling

Eighty male SPF-grade KM mice (weights of 25 ± 5 g) were supplied by the Experimental Animal Center of Lanzhou University (Lanzhou, China). These mice were kept in SPF-grade laboratory conditions with free access to food and water, room temperature of 22 ± 3°C, the humidity of 50 ± 5%, and controlled light-dark cycles (12/12 h). Before the experiment, A*β*_25–35_ was dissolved in ultrapure water to configure a 1.0 mM solution and incubated at 37°C for 7 days to induce A*β*_25–35_ aggregation and enhance neurotoxicity [26].

After seven days, the mice were divided into eight groups using the random number table method: the control check (CK), the AD model group (AD), the sham-operated group (SO), the donepezil treatment group (DON), the husks ethanolic extract high dose treatment group (XANH), the husks ethanolic extract low dose treatment group (XANL), the petroleum ether extract of the seed kernel (PET), and the ethanolic extract of the seed kernel (ETH), with ten mice in each group. The dementia model mice were induced by subcutaneous injection of D-Gal combined with hippocampus injection of A*β*_25–35_, according to reports [27, 28]. The mice were weighed every three days and given the appropriate amount of drug by gavage daily according to the body weight of the mice. The drugs were given according to relevant studies [29]. The gavage dose was 0.01 mL/g/d. Saline was used for the AD, CK, and SO groups. The DON group 0.086 mg/mL of donepezil saline solution. The XANL group received 0.075 mg/mL of ethanolic extract of husks. The XANH group received 0.15 mg/mL of ethanolic extract of husks. The PET group received 0.15 mg/mL of petroleum ether extract of seeds. The ETH group received 0.15 mg/mL of ethanolic extract of seeds. All procedures involving animals were performed according to the ethical guidelines approved by the Lanzhou University Animal Ethics Committee.

### 2.4. Behavior Study

After six weeks of experimentation, the Morris water maze (MWM) test was used to investigate the learning, memory, and spatial exploration ability of different groups of mice [30]. The MWM test consists of two parts, place navigation test and spatial probe test, with a circular pool (120 cm in diameter and 50 cm in height) and a safety platform (6.5 cm in diameter and 16 cm in height) as the experimental setup. The safety platform was placed in the third quadrant, at more than 20 cm from the pool wall, and the water temperature was controlled at 22 ± 2°C. The first day of the MWM study is the platform visibility experiment. The water surface height was adjusted so that the safety platform was 1.0 cm above the water surface, the mice were trained to find the safety platform, the swimming speed of all mice was recorded, and the mice with abnormal locomotor ability were screened out by the swimming speed. On days 2–5 of the experiment for the place navigation investigation, the water surface is higher than the platform 1.5 cm. Each mouse was given four different starting positions for every day of the experiment, and the time it took for the mouse to find and climb onto the platform was the escape latency. The escape latency was recorded for each experiment, if the mouse did not get on the platform within 60 seconds, the escape latency was recorded as 60 seconds. On day six of the MWM study, the safety platform was removed, and the spatial probe test was conducted. Mice were placed in the water from the farthest point in the third quadrant for 60 seconds of spatial exploration, and the time they stayed in the third quadrant was calculated. All data collected in the MWM test were expressed as mean ± SD. The results were statistically analyzed by one-way ANOVA or two-way ANOVA, and *p* < 0.05 indicated statistical significance.

### 2.5. Sample Collection and Preparation

Immediately after the MWM study, the mice were administered the drug by gavage, fasted without water, and executed after 12 hours. The urine was collected and then immediately centrifuged at 3 000 rpm for 10 minutes at low temperature (4°C). The supernatant was separated and stored at −80°C for backup. The urine was removed before the assay and melted at room temperature and centrifuged at 12 000 rpm for 10 minutes at a low temperature (4°C). We took 100 *μ*L of the supernatant, added urease (3 mg/mL) to remove urea, mixed the solution with methanol (800 *μ*L) to precipitate protein, and added ribitol (0.2 mg) for the internal standard. The solution was centrifuged at 12 000 rpm at a low temperature (4°C) for 10 minutes and blown dried using a nitrogen blowing instrument for trimethoprim silane (TMCS) derivatization process [31]. Quality control (QC) samples were prepared by mixing equal aliquots of each urine sample. After derivatization, the supernatant was centrifuged at 13 000 rpm for 5 min and transferred to the autosampler vial for GC/MS analysis.

After the mice were executed to collect urine, whole-brain samples were immediately collected on the icebox. The cerebellum was removed, followed by immediate fixation in 4% paraformaldehyde solution greater than 10 times the volume of tissue for 24 h. Afterward, dehydration transparency, wax immersion, embedding, and coronal sectioning (4 *μ*m) were performed, and hematoxylin-eosin (HE) staining was used on dried paraffin sections to produce HE-stained sections of brain tissue containing the hippocampal region of the mice [32].

### 2.6. GC/MS Analysis

The GC/MS analysis was performed on an Agilent 6890N 5975CGC/MS system (Agilent Technologies, USA). Separation was achieved using the DB-5MS capillary column (30 m × 0.25 m, 0.25 *μ*m, Agilent Technologies, USA) in a split mode (10 : 1). The inlet temperature was 280°C, the carrier gas was high purity helium, and the column flow rate was 1.0 mL/min. The initial GC oven temperature was kept at 70°C for 5 min, with a heating rate 20°C/min to 160°C, 4°C/min to 180°C, and 10°C/min to 300°C, held for 1.5 minutes. MS conditions were as follows. The ionization method is a standard EI source with an electron energy of 70 eV, an ion source temperature of 200°C and a detection voltage of 0.9 kV. MS data were obtained in full-scan mode at a range of *m*/*z* = 35–800 with a scan speed of 5 scans/s. The solvent delay time was 3 min. Six QC samples and one blank sample had been tested before the urine samples were tested. One additional QC sample was taken after every five urine samples. Two more QC samples were tested after the end of the urine sample testing. GC/MS system stability was assessed by QC samples.

### 2.7. Data Processing and Statistical Analysis

GC/MS data were acquired and viewed by MSD ChemStation (Agilent Technologies, USA) and exported to comma delimited format (CDF) files, deconvolved using AMDIS 32.0 (NIST, USA), and then searched for internal standard peaks by mass spectrometry through Nist 14.0 spectral library. We used the Analysis Base File Converter (Reifycs, USA) to convert CDF files into analysis base files (ABF) and imported them into MS-DIAL (CompMS, Japan) for baseline correction, noise reduction, peak alignment, peak area, and standard internal normalization. We then processed the zero values according to the “80% rule” to process zero values [33]. Data were imported into SIMCA-P 14.0 (Umetrics, Sweden) for principal component analysis (PCA) and orthogonal partial tiniest squares analysis (OPLS-DA) at the end of data preprocessing. *T*-tests or one-way ANOVA was performed between subgroups using MetaboAnalyst 5.0 (https://www.metaboanalyst.ca/) to screen data with a VIP > 1 and *p* < 0.05 for potentially differential metabolites, and the Nist 14.0 spectral library was used to search and characterize differential metabolites. Afterward, the potential differential metabolites were imported into MetaboAnalyst 15.0 for metabolic pathway analysis (pathway library: *Mus musculus* KEGG). Pathway impact values were derived after topological analysis. Pathway impact > 0.02 was considered as a potential target metabolic pathway.

## 3. Results and Discussion

### 3.1. The Results of the MWM Experiment

**Figure 1:**
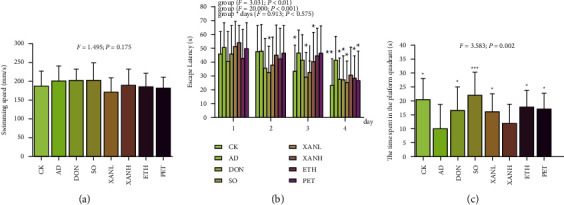
(a), (b), and (c) are the MWM test results of the CK, SO, AD, DON, XANH, XANL, ETH, and PET groups (*n* = 8–10, $\bar{x}$ ± sd, ^*∗*^*p* < 0.05, ^*∗∗*^*p* < 0.01, ^*∗∗∗*^*p* < 0.001 vs. AD group). (a) The result of the platform visible period test in mice, which shows the swimming speed of all mice. (b) The result of the MWM place navigation test in mice, which shows the change of the escape latency of all mice. (c) The result of the spatial probe test, which exhibits the difference of the time mice stayed in the third quadrant.

The results of the MWM experiment for eight groups of mice are shown in Figure 1. The swimming speed of mice in each group during the visible platform period (Figure 1(a)) was not statistically significantly different (*p* < 0.05), indicating that the difference in behavioral test results was not due to differences in motor function outside of cognitive function. The escape latency of all mice in the place navigation test gradually decreased with the increase of experimental days (Figure 1(b)), However the latency of the AD group was still significantly more than the other experimental groups, indicating that the establishment of the AD model was successful. All experimental groups, except the XANH group, showed a significant decrease in latency on days three to four of the experiment (*p* < 0.05). On the final day of the spatial probe test, the AD group mice showed a significant decrease in the time spent in the platform quadrant. Similarly, all groups except the XANH group showed a significant increase (*p* < 0.05) in the platform quadrant retention time compared with the AD group (Figure 1(c)). The experimental results showed that the extract of *Xanthoceras sorbifolia* could improve the learning and memory impairment caused by A*β*_25–35_ and D-Gal, and the improvement effect was related to the saponin content in the drug.

### 3.2. Pathological Changes

**Figure 2:**
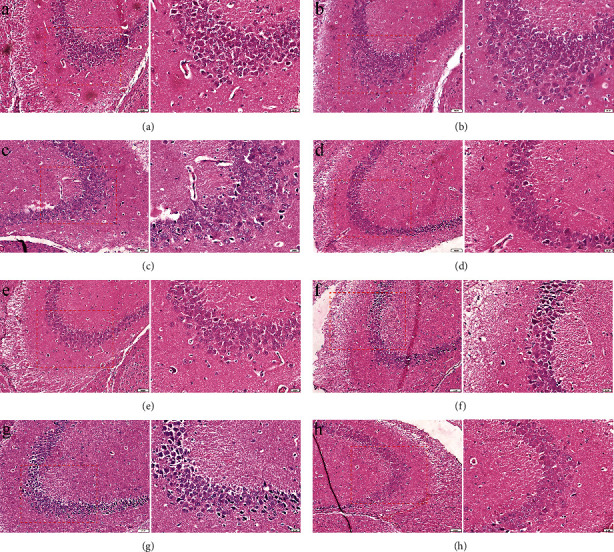
The H & E staining images of the CA1 region of hippocampus (left panel × 200 with insets of scale bar = 50 *μ*m; right panel × 400 with insets of scale bar = 20 *μ*m); (a) the CK group; (b) the SO group; (c) the DON group; (d) the AD group; (e) the ETH group; (f) the PET group; (g) the XANL group; (h) the XANH group.

During the progression of AD, the CA1 pyramidal neurons of the hippocampus are vulnerable to neuronal degeneration [34]. Therefore, this experiment was conducted to study the effect of *Xanthoceras sorbifolia* extract on the mouse AD model by observing the changes of CA1 area in the hippocampus. As shown in Figure 2, the hippocampal tissue of normal mice had an intact cell morphology, neat arrangement, clear cell membranes, no cell pyknosis, nuclear hyperchromasia, and vacuolar degeneration (Figure 2(a)). In contrast, the hippocampal tissue of the AD model group showed blurred cell boundaries, a large number of pyknosis, nuclear hyperchromasia, some vacuolar degeneration, and a small number of cells with lightly stained nuclei and disordered cell arrangement (Figure 2(d)). All pathological sections, except the CK group, showed varying degrees of cell pyknosis, nuclear hyperchromasia, vacuolar degeneration, and faint staining. In contrast, the SO group showed the least lesions in the brain, mainly cell pyknosis and nuclei faint staining (Figure 2(b)), which showed a significant reduction of neuronal damage in the CA1 region of the hippocampus in the SO group. The drug intervention groups showed looser neuronal arrangement and more abnormalities than the SO group, especially in the XANH group (Figure 2(h)), and the results of the histological examination were consistent with the behavioral experiment, indicating that the AD model was successfully established and that the CA1 area of AD mice was protected by the extracts of *Xanthoceras sorbifolia* and donepezil, suggesting that a high dose of *Xanthoceras sorbifolia* husks extract may have a poor effect on the improvement of learning and memory in the AD mice model.

### 3.3. Metabolic Profiling

**Figure 3:**
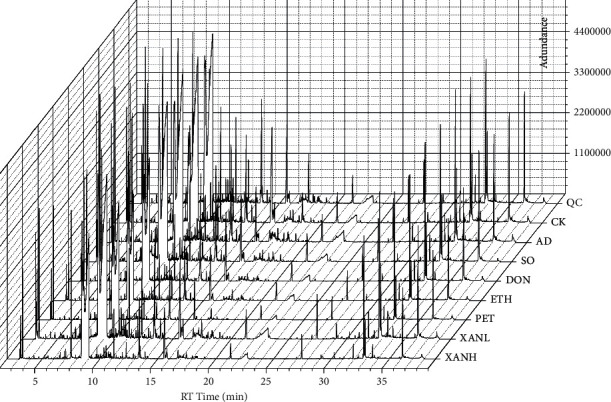
The total ion chromatogram (TIC) of each group's samples after GC/MS analysis.

**Figure 4:**
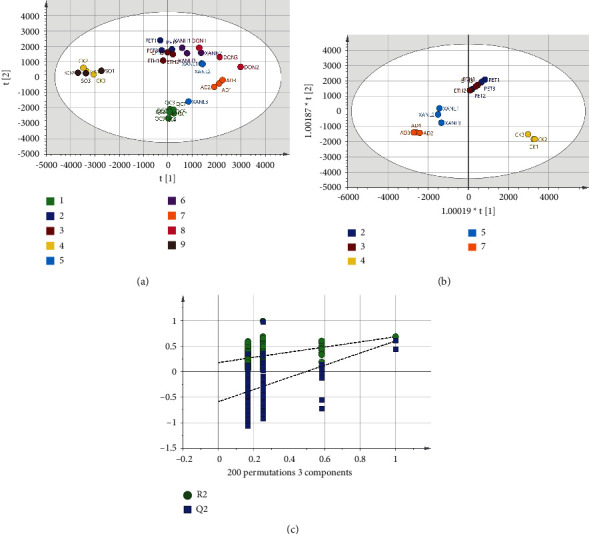
(a) The metabolomic PCA score plot of mouse urine samples from the QC, PRT, ETH, CK, XANL, XANH, AD, DON, and SO groups. (b) The OPLS-DA score plot for the PET, ETH, CK, XANL, and AD groups. (c) The results of OPLS-DA permutation test (*n* = 200). In the figure, the green, deep blue, coffee, yellow, blue, purple, orange, red, and brown dots correspond to the QC, PET, ETH, CK, XANL, XANH, AD, DON, and SO groups.

The total ion chromatogram (TIC) of each group's urine samples after GC/MS analysis is shown in Figure 3. After Pareto (Par) scaling of GC/MS data in SIMCA-P software, the performance of the different experimental groups was analyzed using unsupervised clustering principal component analysis (PCA) with *R*^2^*X* = 0.629 and *Q*^2^ = 0.404 when two components were calculated. In the PCA score plot in Figure 4(a), the QC samples are well aggregated, indicating a stable experimental system. Although some samples overlapped in the PCA score plot, a clear separation occurred between the AD group and the CK and SO groups, indicating significant changes in urinary metabolites in AD model mice. To further distinguish the factors that cause differences between groups and find the most relevant differential metabolites, an unsupervised OPLS-DA model was developed. The CK, AD, and XANL groups are almost completely separated in the OPLS-DA score plot in Figure 4(b). Still, the ETH and PET groups are poorly separated, which may be related to similar mechanisms of action in the two drugs. The OPLS-DA model *R*^2^*X* = 0.854, *R*^2^*Y* = 0.63, and *Q*^2^ = 0.402 was proven to be free of overfitting by the permutation test (*n* = 200) in Figure 4(c).

### 3.4. Differential Metabolite Identification

**Figure 5:**
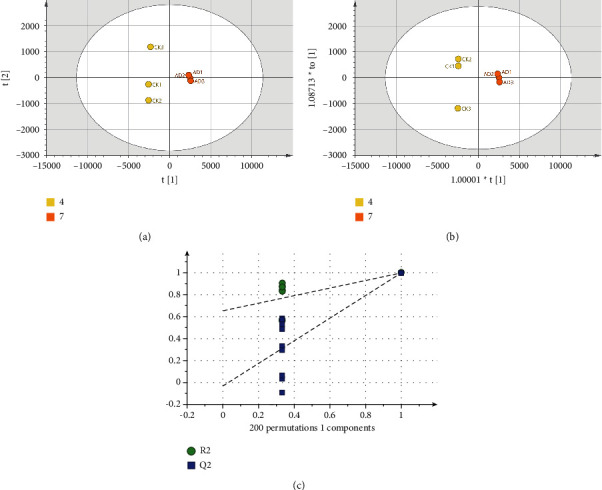
(a) The PCA score plot of urine samples from mice in CK and AD groups. (b) The score plot of OPLS-DA for CK and AD groups. (c) The result of OPLS-DA permutation test (*n* = 200) for CK and AD groups. In the figure, the yellow and orange dots correspond to the CK and AD groups.

To better show the changes in the urinary metabolome after A*β*_25–35_ combined and D-Gal induced dementia model in AD mice, PCA and OPLS-DA analyses were performed using the AD and CK groups. In the PCA score plot (Figure 5(a)) and OPLS-DA (Figure 5(b)), these two groups are clearly separated, with *R*^2^*X* = 0.958 and *Q*^2^ = 0.823 in PCA. The OPLS-DA model showed *R*^2^*X* = 0.902, *R*^2^*Y* = 0.998, and *Q*^2^ = 0.996. The model was proven free of overfitting by the permutation test (*n* = 200), as shown in Figure 5(c). To determine the differential metabolites between the two groups, we searched for potential biomarkers in the urine of AD model mice, obtained variable importance in projection (VIP) using OPLS-DA, and screened metabolites with a VIP > 1.0. Using *t*-test, *p* < 0.05 indicated potential differential metabolites. Differential metabolites were identified by searching the NIST 14 database based on retention times. The results, as shown in Table 1, revealed that levels of malic acid, citric acid, hippuric acid, palmitic acid, N6-acetyl-L-lysine, and stearic acid in the urine of AD mice were significantly lower than those in the CK group. D-mannitol level in the AD group was significantly higher than that in the CK group.

**Figure 6:**
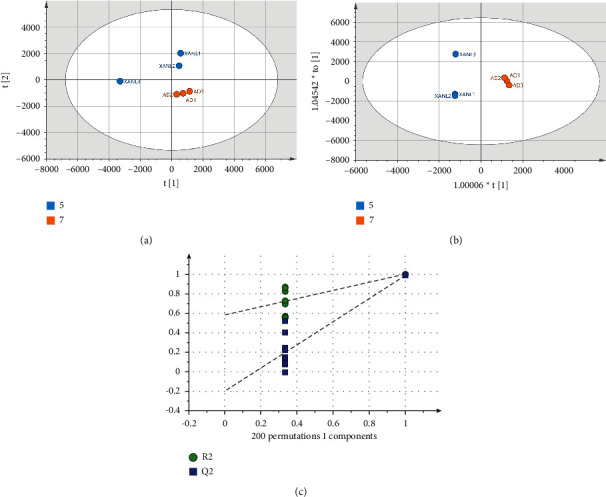
(a) The PCA score plot of urine samples from mice in AD and XANL groups. (b) The score plot of OPLS-DA for AD and XANL groups. (c) The results of OPLS-DA permutation test (*n* = 200) for AD and XANL groups. In the figure, the deep blue and orange dots correspond to the XANL and AD groups.

To investigate the differences in urinary metabolites in AD mice after treatment with extracts from husks of *Xanthoceras sorbifolia*, PCA and OPLS-DA models were established using the AD and XANL groups (Figures 6(a) and 6(b)). *R*^2^*X* = 0.848, *R*^2^*Y* = 0.989, and *Q*^2^ = 0.924 in the OPLS-DA model, and the OPLS-DA model was verified to be free of overfitting by 200 permutation tests (Figure 6(c)). The data were screened by OPLS-DA and *t*-test for a VIP > 1.0 and *p* < 0.05, and the results, as shown in Table 2, revealed that N6-acetyl-L-lysine, stearic acid, and oxoproline levels were significantly higher in the XANL group compared with the AD group, while the levels of 3-hydroxyphenylacetic acid and D-mannitol were significantly increased in the AD group.

**Figure 7:**
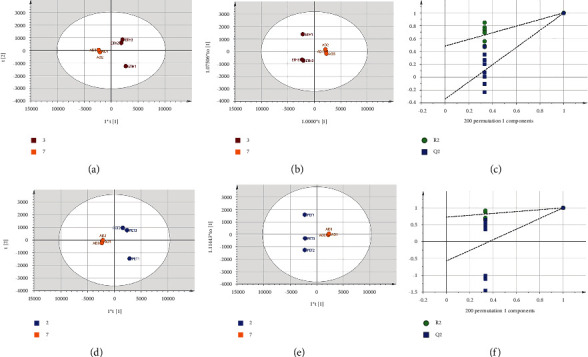
The deep blue, coffee, and orange dots correspond to the PET, ETH, and AD groups. (a) The PCA score plot of urine samples from mice in AD and ETH groups. (b) The OPLS-DA score plot of AD and ETH groups. (c) The result of CK and AD groups' OPLS-DA permutation test (*n* = 200). (d) The PCA score plot of urine samples from mice in AD and PET groups. (e) The OPLS-DA score plot of AD and PET groups. (f) The OPLS-DA permutation test (*n* = 200) for AD and PET groups.

To investigate the urinary metabolomic changes between the ETH and PET groups and the AD group, PCA and OPLS-DA analyses were performed using the ETH and AD groups and the PET and AD groups, separately. The score plots for unsupervised PCA and supervised OPLS-DA (Figure 7) show a clear separation. The *R*^2^ and *Q*^2^ in the OPLS-DA model for both groups were greater than 0.5, indicating the good predictive ability of the model, and the model was verified to be free of overfitting by the permutation test (*n* = 200) as shown in Figures 7(c) and 7(f). The data were screened by OPLS-DA and *t*-test for a VIP > 1.0 and *p* < 0.05, and the results as shown in Table 3 were obtained. Compared with the AD group, the urinary levels of citric acid, pentanedioic acid, stearic acid, malic acid, and palmitic acid were significantly elevated in the ETH group, while the levels of N6-acetyl-L-lysine, taurine, and 2-propanamine were decreased considerably. As shown in Table 4, the levels of citric acid, stearic acid, D-gluconic acid, and palmitic acid in the urine of mice in the PET group were significantly increased, while the levels of N6-acetyl-L-lysine, taurine and gulonic acid were significantly decreased. The results of the differential metabolite analysis showed that the use of seed kernel extracts from *Xanthoceras sorbifolia* treated with different methods produced similar effects on urinary metabolites in AD model mice, suggesting that the two drugs may have the same mechanism of action in AD mice.

### 3.5. Metabolic Pathway Analysis

**Figure 8:**
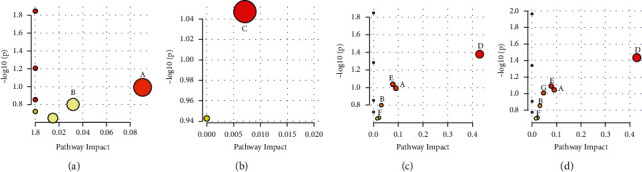
The bubble map of urinary metabolic pathways in all mice. (a) AD and CK groups; (b) AD and XANL groups; (c) AD and ETH groups; (d) AD and PET groups. *A* is the citrate cycle; *B* is the glyoxylate and dicarboxylate metabolism; *C* is the glutathione metabolism; *D* is the taurine and hypotaurine metabolism; *E* is the pentose and glucuronate interconversions; *F* is the primary bile acid biosynthesis; *G* is the pentosephosphate pathway.

The differential metabolites analyzed from each group were imported into MetaboAnalyst 15.0 for a metabolic pathway analysis. The analysis results as shown in Figure 8 indicate that the possible metabolic pathways in order of pathway impact from the highest to lowest are as follows: (1) AD and CK groups: citrate cycle, glyoxylate, and dicarboxylate metabolism; (2) AD and XANL groups: glutathione metabolism; (3) AD and ETH groups: metabolism of taurine and hypotaurine, tricarboxylic acid cycle, pentose, and glucuronic acid; (4) AD and ETH groups: taurine and hypotaurine metabolism, citrate cycle, pentose and glucuronate interconversions, pentose phosphate pathway, metabolism of glyoxylate and dicarboxylic acid, and primary bile acid biosynthesis.

Experimental studies have shown that oligomers of A*β* in the brain are more neurotoxic compared with A*β*-deposited plaques [35]. Neurons in the CA1 region of the hippocampus are susceptible during AD progression, D-Gal induces stable aging effects in animals, and studies have shown that arterial injection of A*β* in mice causes memory impairment, leading to oxidative stress and neuronal damage [36]. Therefore, the animal model established by using hippocampal CA1 injection of A*β*_25-35_ combined with subcutaneous injection of D-Gal can be regarded as an alternative to the transgenic animal model, and the water maze experiment also showed that the animal model established by A*β*_25–35_ combined with subcutaneous injection of D-Gal could significantly induce learning and memory dysfunction.

Based on GC/MS urine metabolomics approach, we found seven potential biomarkers from the urine of AD model mice. Among them, the levels of malic acid and citric acid, which are involved in the citric cycle (TCA cycle) significantly decreased. The brain metabolizes glucose and oxygen at a reduced rate during the pathogenesis of AD. TCA cycle generates ATP by converting glucose into reducing equivalents of NADH [37]. During the progression of AD, the TCA cycle is inhibited by a surge in energy demand in mice, probably due to the inflammatory response, which consumes large amounts of citric and malic acids. The altered TCA cycle is closely related to brain metabolic rate and free radical production [38]. AD patients have significant impairment of multiple enzymes involved in the TCA cycle [39], leading to abnormal levels of metabolites associated with the TCA cycle. In contrast, a significant increase in citric acid and malic acid levels could be seen in the urinary metabolomic analysis of *Xanthoceras sorbifolia* seed extract, suggesting that *Xanthoceras sorbifolia* seed extract may have improved learning and memory impairment in AD model mice by enhancing TCA circulation, increasing brain metabolic rate, and reducing free radical production. These effects may be attributed to the triterpenoid xanthoceraside, which has been shown to reverse the abnormally low ATP levels in the mouse cerebral cortex and hippocampus and inhibit the impairment of critical enzymes in the TCA cycle from promoting mitochondrial function [16]. On the other hand, citric acid is involved in both glyoxylate and dicarboxylic acid metabolism, which are closely related to oxidative reactions and can affect the levels of various reactants in the TCA cycle [40], suggesting that oxidative reactions and energy metabolism are significantly altered in AD patients.

Neuroinflammation is considered a significant cause of AD as a common pathway of blood-brainbarrier-mediated central nervous system diseases [41]. Stearic acid and palmitic acid contents in the urine of AD model mice were significantly decreased, while a significant increase in stearic acid and palmitic acid contents occurred after treatment with seed kernel extracts of *Xanthoceras sorbifolia*. Studies have shown the presence of stearic and palmitic acids to induce NF-*κ*B activity with potential antioxidant and anti-inflammatory abilities [42]. The seed extracts of *Xanthoceras sorbifolia* may have improved learning and memory impairment in mice by producing an inhibitory effect on inflammatory responses induced by A*β* and inflammatory cytokines, and this change may be related to the presence of anti-inflammatory compounds such as flavonoids, propiophenols, and catechins in *Xanthoceras sorbifolia* [43].

Among amino acid metabolism, the study observed differences in glutathione metabolism in the XHL group and differences in taurine and hypotaurine metabolism in the PET group, both of which are closely associated with aging [44]. 5-Hydroxyproline is an end product of glutathione metabolism. Glutathione metabolism can neutralize peroxyl radicals, which is important for maintaining the homeostasis of reactive oxygen species in the body [45]. The changes in glutathione metabolism within AD group may suggest an imbalance in redox homeostasis, while extracts of *Xanthoceras sorbifolia* husks may protect the homeostasis of reactive oxygen species in vivo by scavenging free radicals through the protection of glutathione metabolism, and the antioxidant activity of *Xanthoceras sorbifolia* husks extracts may be closely related to the high content of xanthoceraside in husks. D-gluconic acid is involved in pentose phosphate pathway (PPP), and the oxidative branch of PPP plays an important role in antioxidation [46], The urinary levels of D-glucuronide were significantly higher in the PET group, and there may be an enhancement of PPP to achieve the inhibitory effect on AD through this pathway.

It was also observed that the improvement of AD in the PET group may be related to bile acid metabolism, taurine is involved in primary bile acid biosynthesis, and increased urinary taurine levels in the AD model may be associated with diminished bile acid metabolism. Bile acid metabolism can affect glucose and lipid metabolism, and there is an association with the development of AD and type 2 diabetes [47], but the exact mechanisms need to be further investigated.

## 4. Conclusions

In conclusion, we investigated the potential ameliorative effect of *Xanthoceras sorbifolia* extracts on AD by performing a nontargeted metabolomics study. After the establishment of AD mouse model, based on GC/MS technique, combined with multivariate analysis and other statistical methods, the metabolomic analysis of mouse urine was performed, and the experimental results showed significant differences between groups in the urine metabolic profiles of mice, indicating different intervention mechanisms of different extracts of *Xanthoceras sorbifolia* corniculatus on AD mice. Fourteen biomarkers with significant differences were identified by screening, and potential metabolic pathways were outlined. We conclude that energy metabolism, fatty acids, neuroinflammatory response, glucose metabolism, and bile acid metabolism play significant roles in the improvement of AD using *Xanthoceras sorbifolia* extracts. We revealed the ameliorative effect of *Xanthoceras sorbifolia* extracts on AD from the metabolic pathways.

## Figures and Tables

**Table 1:** The differential metabolites between the AD and CK groups.

Differential metabolites	RT/min	Fold (AD/CK)
N6-acetyl-L-lysine	9.745	0.33
Malic acid	10.337	0.46
Citric acid	15.628	0.27
Hippuric acid	15.843	0.77
D-Mannitol	18.267	1.47
Palmitic acid	21.314	0.75
Stearic acid	27.44	0.84

**Table 2:** The differential metabolites between the AD and XANL groups.

Differential metabolites	RT/min	Fold (AD/XANL)
Oxoproline	6.866	0.17
N6-Acetyl-L-lysine	9.745	0.62
3-Hydroxyphenylacetic acid	11.915	0.64
D-Mannitol	18.267	0.49
Stearic acid	27.44	2.06

**Table 3:** The differential metabolites between the AD and ETH groups.

Differential metabolites	RT/min	Fold (AD/XANL)
2-Propanamine	4.391	1.37
N6-Acetyl-L-lysine	9.647	1.06
Malic acid	10.337	0.53
Pentanedioic acid	11.205	0.52
Taurine	12.285	1.34
Citric acid	15.628	0.49
Palmitic acid	21.314	0.40
Stearic acid	27.44	0.44

**Table 4:** The differential metabolites between the AD and PET groups.

Differential metabolites	RT/min	Fold (AD/XANL)
N6-Acetyl-L-lysine	9.647	1.45
Taurine	12.285	1.31
Citric acid	15.572	0.75
Gulonic acid	18.004	1.21
D-Gluconic acid	19.028	0.93
Palmitic acid	21.272	0.47
Stearic acid	27.44	0.58

## Data Availability

The data that support the findings of this study can be obtained from the corresponding author upon reasonable request.

## Supplementary Materials

Supplementary Materials The detailed process of total saponin measuring, modeling, drug administration, and behavior study is illustrated in the Supplementary Materials.
