# Supplementary material for: GC/MS-Based Urine Metabolomics Study on the Ameliorative Effect of Xanthoceras sorbifolia Extract on Alzheimer's Disease in Mice
Source: Evid Based Complement Alternat Med. 2022 Sep 17;2022:3390034. doi: 10.1155/2022/3390034 (PMC9509262; doi:10.1155/2022/3390034)
Supplement: Supplementary Materials — The detailed process of total saponin measuring, modeling, drug administration, and behavior study is illustrated in the Supplementary Materials. [file 3390034.f1.pdf]

Supplementary material

Determination of the total saponin content of the Xanthoceras Sorbifolia extract

Ginsenoside Re was used as the standard, and the standard curve was plotted by spectrophotometry and the regression equation was calculated, after which the total saponin content in the extract was further determined by spectrophotometry. Weighed 10 mg ginsenoside Re, dissolved completely with 3-5 mL anhydrous ethanol and transferred to a 25 mL volumetric flask, fixed the volume of anhydrous ethanol to the scale and shaken well to obtain 0.4 mg/mL of life saponin Re standard solution. Then add 0.2 mL of newly configured 5% vanillin-glacial acetic acid solution and 0.8 mL of perchloric acid, heat up the water bath at 70 ℃ for 15 min, add 5 mL of glacial acetic acid and mix well, and let it stand for 15 min. The spectrophotometer was immediately zeroed with distilled water and the absorbance at 546 nm was measured. With the mass of ginsenoside Re as the horizontal coordinate and absorbance as the vertical coordinate, the standard curve was plotted and the regression equation and coefficient of determination were calculated. The experimental data are shown in Table 1, and the standard curve is shown in Figure 1.

Table 1 Ginsenoside re standard solution determination results

Serial number	Ginsenoside Re mass (mg)	Absorbance
1	0.04	0.290
2	0.08	0.547
3	0.12	0.761
4	0.16	0.998
5	0.2	1.207

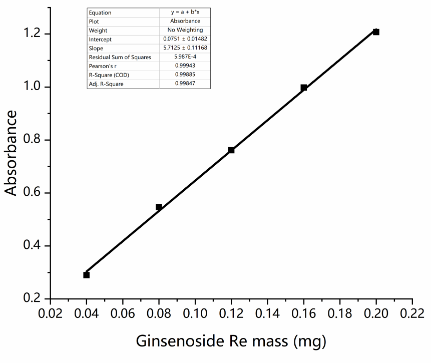

Figure 1 Ginsenoside Re standard curve. Regression equation: Y = 5.7125 x+0.0751,coefficient of determination: R2 = 0.9989.

Three portions of the dried powder of the husks extract and 5.0 mg of the petroleum ether extract of the seed kernel were weighed and dissolved completely in 3-5 mL of anhydrous ethanol and transferred to a 25 mL volumetric flask, and the sample solution of 0.2 mg/mL was obtained by shaking well. Use a pipette to transfer 0.2 mL and 0.4 mL of the sample solution into a 10 mL stoppered test tube, water bath to completely evaporate the solvent, and then add 0.2 mL of newly configured volume fraction of 5% vanillin-glacial acetic acid solution and 0.8 mL of perchloric acid, heat up the water bath at 70 °C for 15 min and then continue to add 5 mL of glacial acetic acid vortex mix, let stand for 15 min and then immediately use the spectrophotometer distilled water The absorbance at 546 nm was measured. After obtaining the absorbance of 12 samples, the average absorbance of each sample at different concentrations was calculated separately, and the mass of saponins in the samples was obtained using this value brought into the regression equation, and the saponin content was calculated from the sample saponin mass, and the total sample saponin content (%) = total saponin mass / sample mass × 100. the results of the experiment are shown in Table 2.

Table 2 Determination results of total saponins from the husks and seed kernel extract

Experiment number	Absorbance (A)		Total saponin mass(mg)		Total saponin content (%)
	0.04 mg	0.08 mg	0.04 mg	0.08 mg	
1	0.233±0.0024	0.396±0.0021	0.028	0.056	69.75
2	0.183±0.0078	0.269±0.0043	0.020	0.034	46.07

Note: Experiment No. 1 is the experiment of determination of ethanol extract of the husks ; experiment No. 2 is the experiment of determination of petroleum ether extract of seed kernels.

Establishment of mouse AD model

Mice were anesthetized by intraperitoneal injection with 1.25% tribromoethanol (avertin) solution (0.2 mL/10g), and the surgical area of the scalp was debrided and prepared using ophthalmic scissors and fixed on a brain stereotaxic instrument. The surgical area was disinfected with iodophor disinfectant, and the scalp was incised to expose the fontanelle. The location of the hippocampal CA1 area was confirmed, and the positioning of the injection site was performed. Using fontanel as a reference, the right hippocampal area was injected: anterior-posterior: - 2.3 mm, left-right: + 1.8 mm, and the skull was drilled with a dental slow-speed handpiece at the above positioning point. Except for the control and sham-operated groups, each mouse was slowly injected with 5 μL of cohesive $A\beta_{25-35}$ suspension over 5 min using a 10 μL microsampler with a needle depth of 2 mm in the fontanel plane. The needle was then left in place for 5 minutes. Mice in the sham-operated group were positioned in the hippocampus in the same way, but the needle was only left in place for 5 minutes without injection of the drug. The wound was carefully sutured after the injection. To ensure the accuracy of the experiment, the mice were injected with 1% gentian violet solution in the hippocampal region under the brain stereotaxic apparatus before the start of the formal experiment, and the mice were executed immediately after the injection, and the brain tissue was dissected to determine that the injection site was located in the CA1 region of the hippocampus as shown in Figure 2.

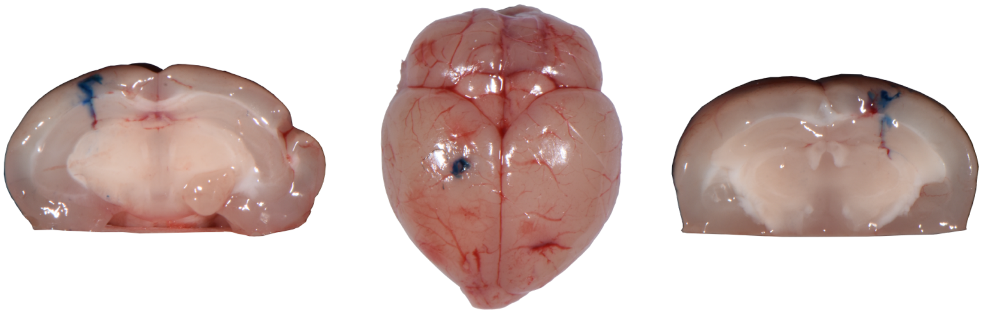

A B C

Figure 2 Distribution of drug after stereotactic brain injection in the CA1 region of the hippocampus in mice.

The Morris water maze test

Day 1 of the experiment was a platform-visible experiment, where mice were trained to find a safe platform and kept in the behavioral laboratory 10 hours in advance for environmental adaptation. The structure of the water maze is shown in Figure 3. The pool is divided into four quadrants, and the platform is located in quadrant E with a height of 1 cm above the water surface, keeping the pool water transparent and placing black markers on the platform to ensure that mice can see the platform in the water. First, the mice were placed on the safety platform for 20 seconds, and then the mice were gently held by hand in order to place them into the water at positions 1, 2, 4 and 5 in the figure for four experiments respectively. Mice were allowed to explore freely in the water for 1 minute to find a safe platform, and the search for the platform was considered successful if the mice reached the platform and stopped for 10 seconds within 1 minute. If no platform was found for more than 1 minute, the mice were manually guided to a safe platform and allowed to stay on the platform for 15 seconds or longer. After completing the experiment, the mice were immediately fished out of the pool and dried with a towel and placed in a small mouse cage next to the heater. The mice were trained to find a safe platform, and the swimming speed of all mice was collected. According to the experimental results, mice with slow swimming speed due to motor organ injury, those that were stationary in the water for a long time and those that swam too fast were removed and then the subsequent experiments were conducted.

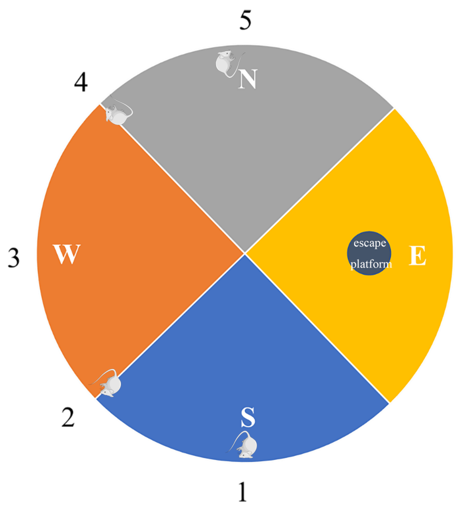

Figure 3 Construction of water maze facilities

The positioning navigation experiment was started on day 2 of the experiment, and the safety platform was still located in quadrant E as shown in Figure 3. At this time, titanium dioxide was poured into the water, mixed well, and the pool became completely turbid, and the platform was allowed to lie 0.5 cm below the water surface to ensure that the safety platform could not be seen when the mice were in the water. Boarding the safety platform within 1 minute and holding it for 10 seconds was

considered successful. If the platform was not found for more than 1 minute, the mice were manually guided to the safety platform and allowed to stay on the platform for 15 seconds before continuing to the next experiment. The mice were kept for 4 consecutive days, and the time spent to find and climb on the platform was called the latency period. If the mice did not get on the safety platform within 1 minute, the latency period was recorded as 60 seconds, and the latency period of each mouse was recorded and the video data of the whole process was kept.

On the 6th day of the experiment, the spatial exploration experiment was started, and the water maze facility was built as shown in Figure 4. The safety platform in the water maze was removed, and the titanium dioxide turbid pool was used, and the mice were put into the water at the farthest location from the original safety platform, i.e., location 3. The retention time of the mouse in the quadrant of the original platform was recorded within 60 seconds. The partial movement trajectories of all mice performing the MWM experiment are shown in Figure 5.

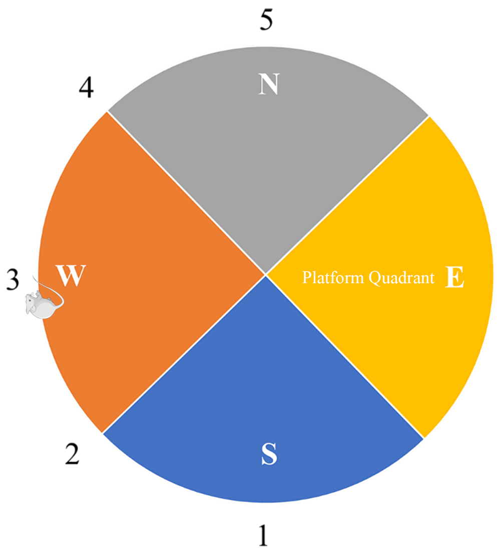

Figure 4 Construction of water maze facilities during the spatial probe test.

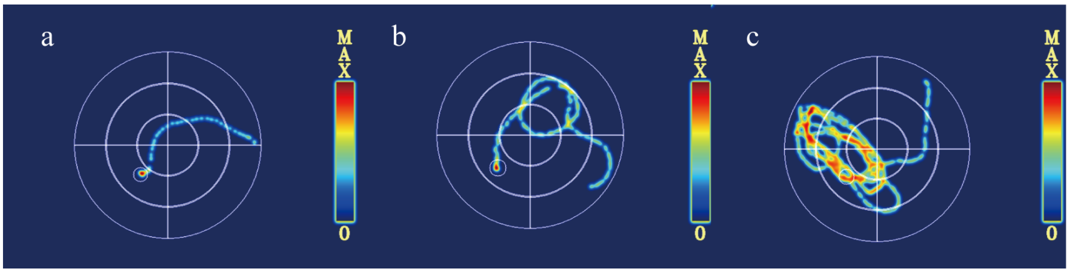

Figure 5 mouse tracks in the MWM test. Figure 5(a) shows the tracks of mice in the platform visibility period; Figure 5(b) shows the tracks of mice in the positioning navigation period; Figure 5(c) shows the tracks of mice in thespatial probe test.
